# Influence of non-pharmaceutical interventions on epidemiological characteristics of *Mycoplasma pneumoniae* infection in children during and after the COVID-19 epidemic in Ningbo, China

**DOI:** 10.3389/fmicb.2024.1405710

**Published:** 2024-07-17

**Authors:** Min Jiang, Hui Zhang, Fangfang Yao, Qinhong Lu, Qian Sun, Zhen Liu, Qingcao Li, Guangliang Wu

**Affiliations:** ^1^Department of Clinical Laboratory, The Affiliated LiHuiLi Hospital of Ningbo University, Ningbo, China; ^2^Department of Clinical Laboratory, Ninghai County Chengguan Hospital, Ningbo, China; ^3^Department of Clinical Laboratory, Ningbo Yinzhou No. 2 Hospital, Ningbo, China; ^4^Department of Clinical Pharmacy, The Affiliated LiHuiLi Hospital of Ningbo University, Ningbo, China

**Keywords:** *Mycoplasma pneumoniae*, children, COVID-19, NPIs, epidemiological characteristics

## Abstract

**Background:**

Since the outbreak of COVID-19, China has implemented a series of non-pharmaceutical interventions (NPIs), effectively containing the spread of severe acute respiratory syndrome coronavirus 2 (SARS-CoV-2) as well as various respiratory pathogens. With the continuous relaxation of restrictions, China has entered a new phase of the post-pandemic era. However, the epidemiological differences of *Mycoplasma pneumoniae* (MP) between the two phases in Ningbo and even in China remain unclear.

**Methods:**

Data of children aged 0–14 years who visited the Ningbo Medical Center LiHuiLi Hospital due to acute respiratory tract infections from January 2020 to December 2023 were collected. PCR was used to detect 13 respiratory pathogens and the macrolide-resistance of *Mycoplasma pneumoniae*.

**Results:**

Among 10,206 children, 2,360 were infected with MP (23.12%). Among the total, the MP positive rate during the NPI phase (6.35%) was significantly lower than that during the non-NPI phase (34.28%), while the macrolide resistance rate increased from 62.5% (NPI phase) to 81.1% (non-NPI phase). The rate of MP co-infection increased from 11.2% (NPI phase) to 30.3% (non-NPI phase). MP infection exhibited obvious seasonality, with the highest prevalence in autumn (30.0%) followed by summer (23.6%). There were differences in MP positivity rates among different age groups, with the highest among school-age children at 39.5%. During the NPI phase, all age groups were less susceptible to MP, while during the non-NPI phase, the susceptible age for MP was 4–12 years, with 8 years being the most susceptible. The susceptible age for MP co-infection was 0–6 years. MP exhibited antagonistic effects against numerous pathogens. Compared to MP single infection, the proportion of pneumonia was higher in MP co-infection cases.

**Conclusion:**

The removal of NPIs significantly impacted the spread of MP, altering population characteristics including age, seasonality, macrolide resistance, and MP co-infection rates.

## 1 Introduction

*Mycoplasma pneumoniae* (MP) is one of the smallest prokaryotic organisms lacking a cell wall, exhibiting characteristics between bacteria and viruses. It can grow on non-living surfaces and is commonly spread through aerosols and droplets, with its infection rate positively correlated with temperature, making it prevalent throughout the year but particularly so during the late summer and early autumn (Leng et al., 2023). MP is a major pathogen causing acute respiratory tract infections in children, leading to symptoms such as fever, cough, pharyngitis, bronchitis, and pneumonia, accounting for 10–40% of community-acquired pneumonia cases (Yi et al., 2022). MP infection typically manifests as a self-limiting disease with mild symptoms, but severe cases can progress to pneumonia and trigger severe extrapulmonary infections, even resulting in mortality (Kuo et al., 2022).

Macrolide antibiotics are the first-line treatment for MP infection. With the widespread use of macrolide antibiotics, macrolide-resistant *Mycoplasma pneumoniae* (MRMP) was first reported in Japan (Okazaki et al., 2001) and quickly spread worldwide. The detection rate of MRMP in the United States and Europe is relatively low (Xiao et al., 2020; Leng et al., 2023), around 10%, while in China and South Korea, it can reach 56–90% (Xiao et al., 2020; Zhou et al., 2020; Wang et al., 2022; Shin et al., 2023; Xu et al., 2024), showing significant regional variations. Studies have shown interactions between pathogens, with MP capable of mixed infections with other pathogens. However, research on co-infection is limited, and whether mixed infections lead to more severe clinical manifestations or prolonged and aggravated courses compared to single MP pathogen infections requires further investigation.

Since 2020, the outbreak of COVID-19 has disrupted the daily lives of individuals worldwide. To limit the spread of the virus, nearly all countries have implemented non-pharmaceutical interventions (NPIs), including wearing masks, maintaining social distancing, enhancing hand hygiene, home isolation, closing entertainment venues, implementing remote education, and restricting travel. NPIs aim to reduce the transmission of respiratory pathogens, including influenza viruses and human respiratory syncytial virus (HRSV), thereby significantly affecting the seasonal epidemic patterns (Chow et al., 2023). Thus far, studies on the epidemiological characteristics of MP infections before and after the lifting of NPIs during the COVID-19 pandemic in Ningbo, China, have been sporadic or even non-existent. This study retrospectively analyzed MP infection data from January 2020 to December 2023, aiming to evaluate the impact of NPIs on the epidemiological characteristics of MP and explore the factors influencing MRMP and MP co-infection, as well as the interactions between pathogens, providing a scientific basis for the monitoring and control of *Mycoplasma pneumoniae* infections in the post-pandemic era.

## 2 Methods

### 2.1 Study subjects

Clinical data of children aged 0–14 years with acute respiratory symptoms (such as cough, rhinorrhea, fever, auscultatory rales in the lungs, wheezing, with or without moist rales) admitted to the Ningbo Medical Center LiHuiLi Hospital from January 2020 to December 2023 were collected. All children underwent testing for *Mycoplasma pneumoniae* and macrolide resistance, as well as 13 respiratory pathogens. Patients with underlying diseases (congenital diseases, immunodeficiency diseases, etc.), recurrent chronic respiratory diseases, or incomplete clinical data were excluded from the study. This study was approved by the Ethics Committee of the Ningbo Medical Center LiHuiLi Hospital (Ethical approval number: KY2024SL070-01).

### 2.2 Grouping

Based on the implementation time of COVID-19 epidemic prevention and control measures, this study was divided into two phases: NPI phase (2020–2022) and non-NPI phase (2023). Children were categorized into four age groups: <1 year (infant group), 1–2 years (toddler group), 3–5 years (preschool group), and 6–14 years (school-age group). According to the month of *Mycoplasma pneumoniae* infection, children were classified into four seasons: spring (March–May), summer (June–August), autumn (September–November), and winter (December–February).

### 2.3 Laboratory testing

Nasopharyngeal specimens (throat swabs, nasal swabs, etc.) were collected from the children for *Mycoplasma pneumoniae* and macrolide-resistant testing (Mole Bioscience, Jiangsu, China) and detection of 13 respiratory pathogens (Haier Gene Technology, Ningbo, China). Macrolide-resistance in *Mycoplasma pneumoniae* was determined by fluorescence PCR, detecting mutations at 23S rRNA positions 2,063 (A:G) and 2,064 (A:G), with the presence of any mutation reported as macrolide resistant. Detection of the 13 respiratory pathogens was performed using PCR capillary electrophoresis fragment analysis, targeting influenza A virus (FluA, includes H7N9, H1N1, H3N2, H5N2), H1N1 (2009), H3N2, influenza B virus (FluB includes Victoria, Yamagata), human parainfluenza virus (HPIV includes 1 through 4 types), human rhinovirus (HRV), bocavirus (Boca), human coronavirus (Hcov, includes 229E, OC43, NL63, and HKU1), human respiratory syncytial virus (HRSV includes A and B groups), human metapneumovirus (HMPV), human adenovirus (HADV includes B, C, and E groups), *Chlamydia* (Ch includes *Chlamydia trachomatis* and *Chlamydophila pneumoniae*), and *Mycoplasma pneumoniae* (MP).

### 2.4 Data analysis

Data analysis was performed using SPSS version 25.0 (IBM Corp., Armonk, NY, United States). Non-normally distributed continuous variables were described using median (interquartile range) and analyzed using the Kruskal–Wallis rank sum test. Categorical data were described using frequencies and percentages, and analyzed using Pearson’s chi-squared test or Fisher’s exact test, as appropriate. Interaction analysis between *Mycoplasma pneumoniae* and other pathogens was conducted using the chi-square test, and odds ratio (OR) was calculated. Differences in clinical diagnosis distribution among different *Mycoplasma pneumoniae* infections were assessed using rank-sum tests. The relationship between age and positivity rate in the NPI phase and non-NPI phase was analyzed using restricted cubic spline (RCS) regression models with four knots, implemented using the R 4.0.5 rms package. A two-sided *p*-value <0.05 was considered statistically significant.

## 3 Results

### 3.1 General clinical characteristics of study subjects

A total of 10,206 cases of acute respiratory tract infection (ARTI) patients were included in this study, with 4,077 cases (39.95%) in the NPI phase (2020–2022) and 6,129 cases (60.05%) in the non-NPI phase (2023). Among this total, 2,360 cases (23.12%) were detected with *Mycoplasma pneumoniae* (MP) infection. The infection rates of MP from 2020 to 2023 were 5.71, 3.03, 10.00 and 34.28%, respectively. There was a significant difference in the MP infection rate between the two phases, increasing from 6.35 to 34.28% (*p* < 0.0001). The detection rate of MP was 22.46% in males and 23.84% in females, with no significant gender difference in MP infection (*p* = 0.1). The outpatient detection rate of MP was 31.65%, significantly higher than the inpatient detection rate of 17.00% (*p* < 0.0001). The median age of the children in this study was 4 years, with a median age of 6 years for MP-positive children, and the median month of MP infection was October. Details are shown in Table 1.

**Table 1:** General clinical characteristics of study subjects.

Characteristics	Overall (*n* = 10,206)	Non-MP (*n* = 7,846)	MP (*n* = 2,360)	*p*-value
**Stage**				<0.0001
NPI phase (2020–2022)	4,077 (39.95%)	3,818 (93.65%)	259 (6.35%)	
NPI phase (2020)	1,050	990 (94.29%)	60 (5.71%)	
NPI phase (2021)	1,487	1,442 (96.97%)	45 (3.03%)	
NPI phase (2022)	1,540	1,386 (90.00%)	154 (10.00%)	
Non-NPI phase (2023)	6,129 (60.05%)	4,028 (65.72%)	2,101 (34.28%)	
**Sex**				0.1000
Male	5,315 (52.08%)	4,121 (77.54%)	1,194 (22.46%)	
Female	4,891 (47.92%)	3,725 (76.16%)	1,166 (23.84%)	
**Type of patients**				<0.0001
Outpatient	4,265 (41.79%)	2,915 (68.35%)	1,350 (31.65%)	
Inpatient	5,941 (58.21%)	4,931 (83.00%)	1,010 (17.00%)	
Age (years)*	4 [2–7]	4 [2–6]	6 [5–8]	<0.0001
Mouth*	9 [6–11]	9 [5–11]	10 [8–11]	<0.0001

“*” Indicated that the Kruskal–Wallis rank sum test was used, and the data was expressed as Median (lQR), the rest was used as Pearson’s chi-squared test, and the data was expressed as *n* (%).

### 3.2 Epidemiological characteristics and seasonal variation of MP

**Figure 1:**
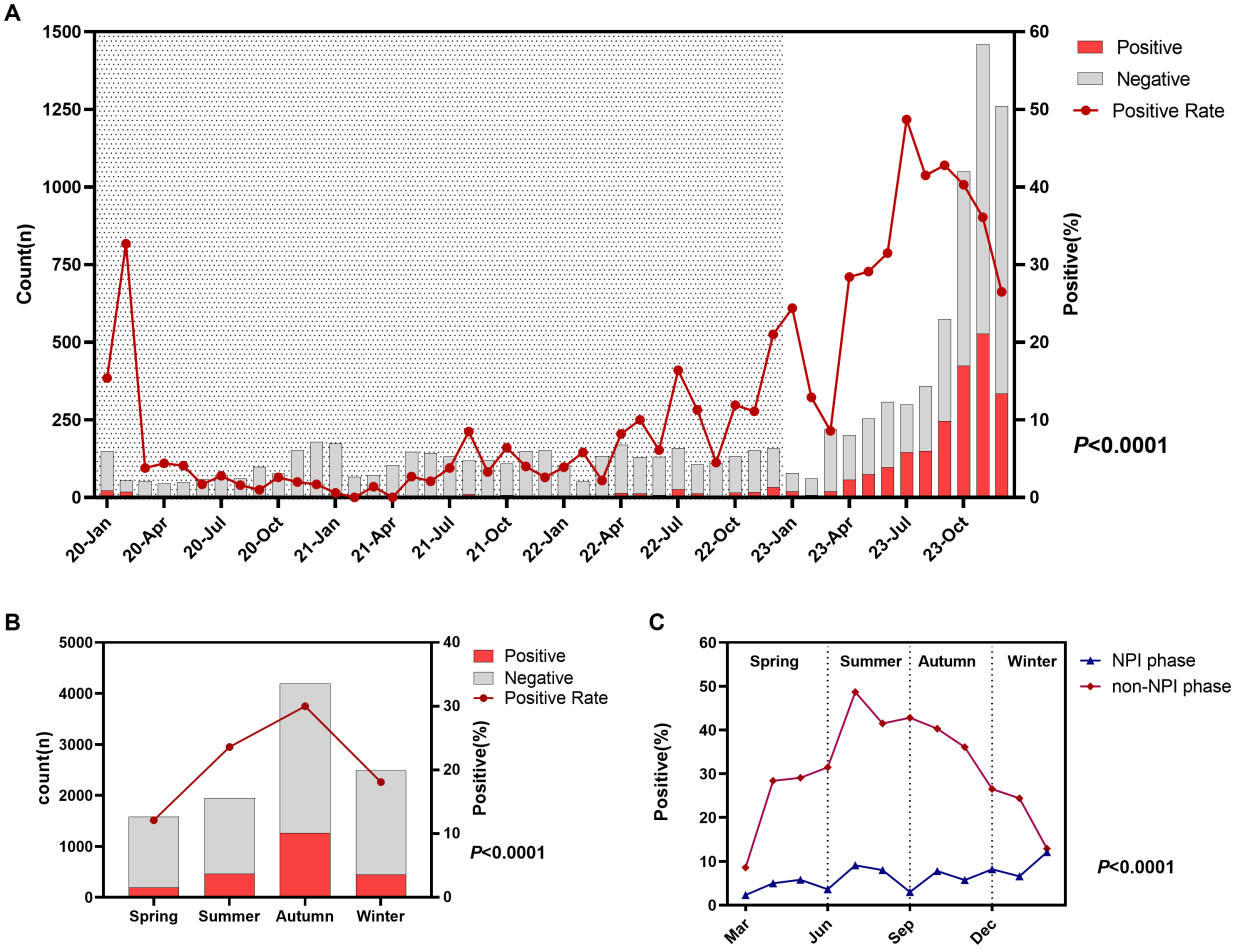
Epidemiological characteristics and seasonal variations of *Mycoplasma pneumoniae*. **(A)** Monthly distribution of acute respiratory tract infection (ARTI) cases and MP positivity rate. Shaded areas represent the NPI phase, while blank areas represent the non-NPI phase. *p*-values indicate the difference in MP positivity rates between the NPI and non-NPI phases. **(B)** Number of ARTI cases and MP positivity rate in different seasons. **(C)** MP positivity rates in different seasons during the two phases. Grey bars represent the number of MP-negative children with ARTI each month, while red bars represent the number of MP-positive children **(A,B)**.

From 2020 to 2022, the number of ARTI children per month was approximately 150, and MP-infected children remained at a low level, with annual positivity rates of 5.7, 3.0, and 10.0%, respectively. In 2023, the number of ARTI children surged, especially from October to December, with an average of over 1,000 cases per month and over 300 MP-infected children per month. The annual positivity rate of MP reached 34.28%, peaking at 48.7% in July. Details are shown in Figure 1A. There was a significant difference in the MP infection rate among different seasons (*p* < 0.0001), with the highest rate in autumn (30.0%), followed by summer (23.6%), winter (18.1%), and spring (12.1%). Details are shown in Figure 1B. To further refine the monthly MP infection rates in the two phases, we plotted Figure 1C. We found that in the non-NPI phase, the MP infection rate in any month was higher than that in the NPI phase, and there was a statistical difference in the MP positivity rate between the two phases (*p* < 0.0001). In the NPI phase, the highest MP positivity rate was 8.2% in winter and the lowest was 4.5% in spring. In the non-NPI phase, the highest MP positivity rate was in summer at 40.5%, followed by autumn at 38.8%, winter at 25.8%, and the lowest in spring at 22.2%.

### 3.3 Detection rate of MP in different age groups

**Figure 2:**
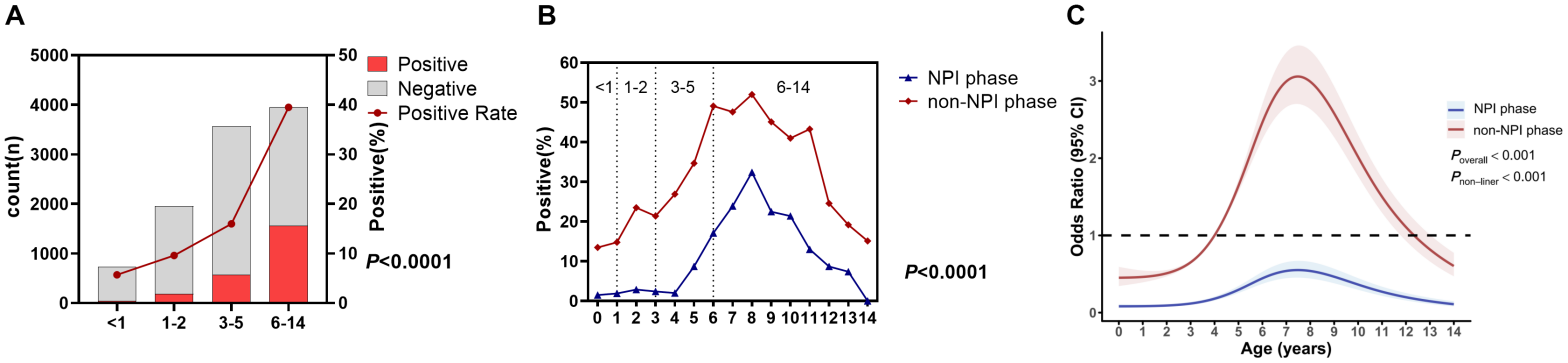
MP positivity rates in different age groups. **(A)** Number of ARTI cases and MP positivity rate in different age groups. Grey bars represent MP-negative children with ARTI, while red bars represent MP-positive children. **(B)** MP positivity rates in different age groups during the two phases. **(C)** Nonlinear analysis of the age-related risk of MP infection during the two phases, with colored shadows representing the 95% confidence interval of the odds ratio (OR).

There was a significant difference in the MP infection rate among different age groups (*p* < 0.0001), with the highest rate in the 6–14 years group at 39.5%, followed by 3–5 years at 16.0%, 1–2 years at 9.6%, and <1 year at the lowest 5.7% (Figure 2A). To further refine the MP infection rates in different age groups in the two phases, we plotted Figure 2B. It was found that in the non-NPI phase, the MP infection rate in any age group was higher than that in the NPI phase, and the age distribution trend was consistent (Figure 2C). Eight years old was a turning point for MP infection. The MP infection rate increased with age from 0 to 8 years and decreased with age from 8 to 14 years. To further understand the relationship between the risk of MP infection and the age of ARTI patients, we conducted unrestricted cubic spline analysis. The risk factors in the non-NPI phase were higher than those in the NPI phase. In the NPI phase, all age groups were not susceptible to MP, while in the non-NPI phase, children aged 4–12 years were susceptible to MP, with 8-year-old children being the most susceptible.

### 3.4 MP antibiotic resistance to macrolides

**Figure 3:**
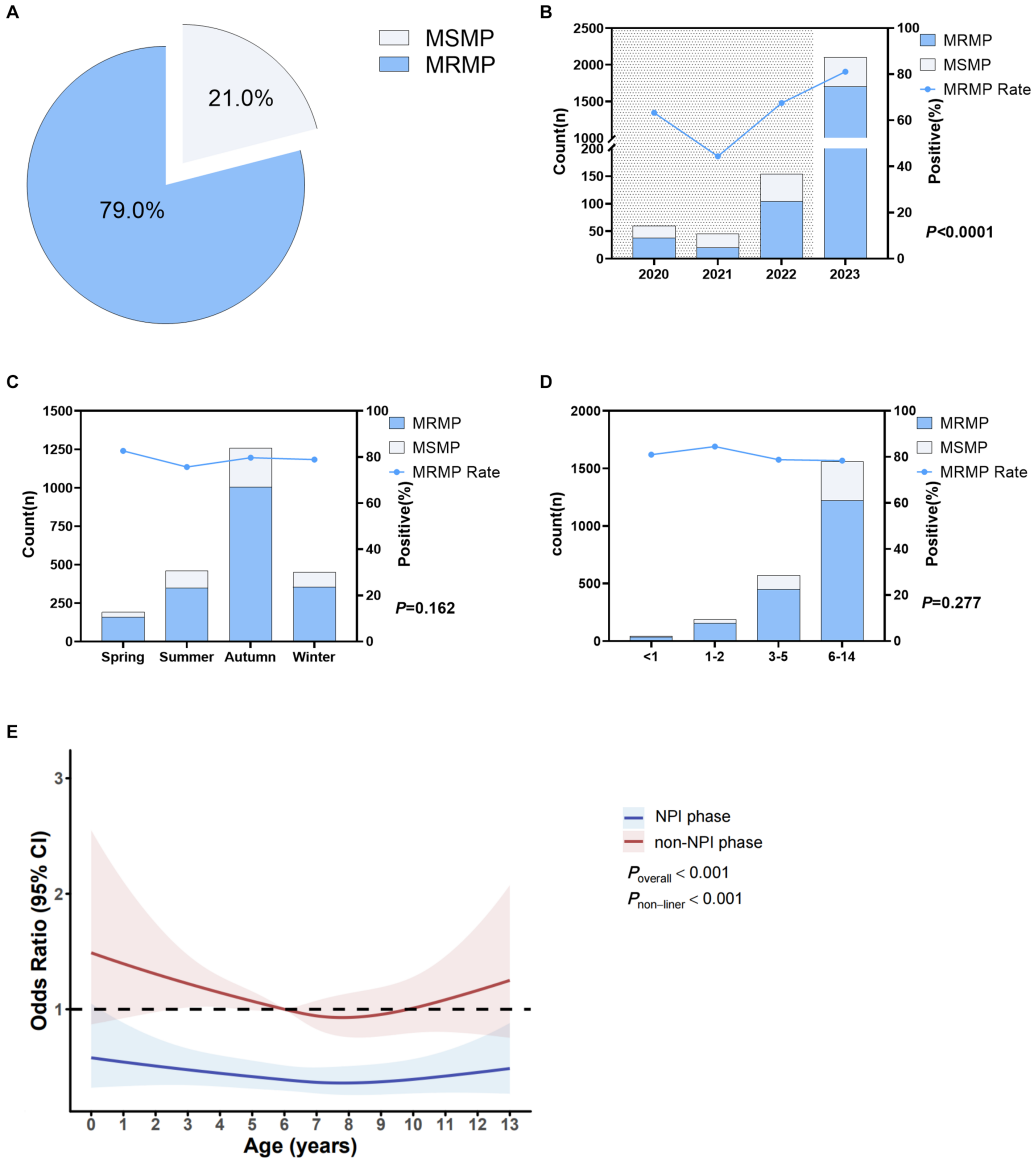
Macrolide resistance in *Mycoplasma pneumoniae*. **(A)** Overall MRMP positivity rate. **(B)** MRMP positivity rates in different years. Shaded areas represent the NPI phase, while blank areas represent the non-NPI phase. *p*-values indicate the difference in MRMP positivity rates between the NPI and non-NPI phases. **(C)** MRMP positivity rates in different seasons. **(D)** MRMP positivity rates in different age groups. *p*-values indicate the difference in MRMP positivity rates between different age groups. **(E)** Nonlinear analysis of the age-related risk of MRMP infection, with colored shadows representing the 95% confidence interval of the odds ratio (OR). Light blue represents the number of macrolide-sensitive MP cases, while blue represents resistant cases **(A–D)**.

In this study, a total of 2,360 cases of MP-positive patients were detected, among which 1,865 cases (79.0%) were resistant to macrolides, and 495 cases (21.0%) were sensitive to macrolides. Details are shown in Figure 3A. Figure 3B shows that the macrolide resistance rates from 2020 to 2023 were 63.3, 44.4, 67.5, and 81.1%, respectively. The macrolide resistance rate in the NPI phase was 62.5%, significantly lower than the resistance rate of 81.1% in the non-NPI phase (*p* < 0.0001). Figure 3C shows that there was no significant difference in the macrolide resistance rate among the four seasons, with resistance rates ranging from 75.7 to 82.7%. Figure 3D indicates that the macrolide resistance rates in children aged <1 year, 1–2 years, 3–5 years, and 6–14 years were 81.0, 84.5, 78.8, and 78.4%, respectively, with no significant difference among age groups (*p* = 0.277). Non-restricted cubic spline analysis revealed that the risk factors in the non-NPI phase were higher than those in the NPI phase (Figure 3E). For children under 8 years old, the MRMP infection rate decreased with age, while for patients over 8 years old, the MRMP infection rate increased with age.

### 3.5 MP co-infection

**Figure 4:**
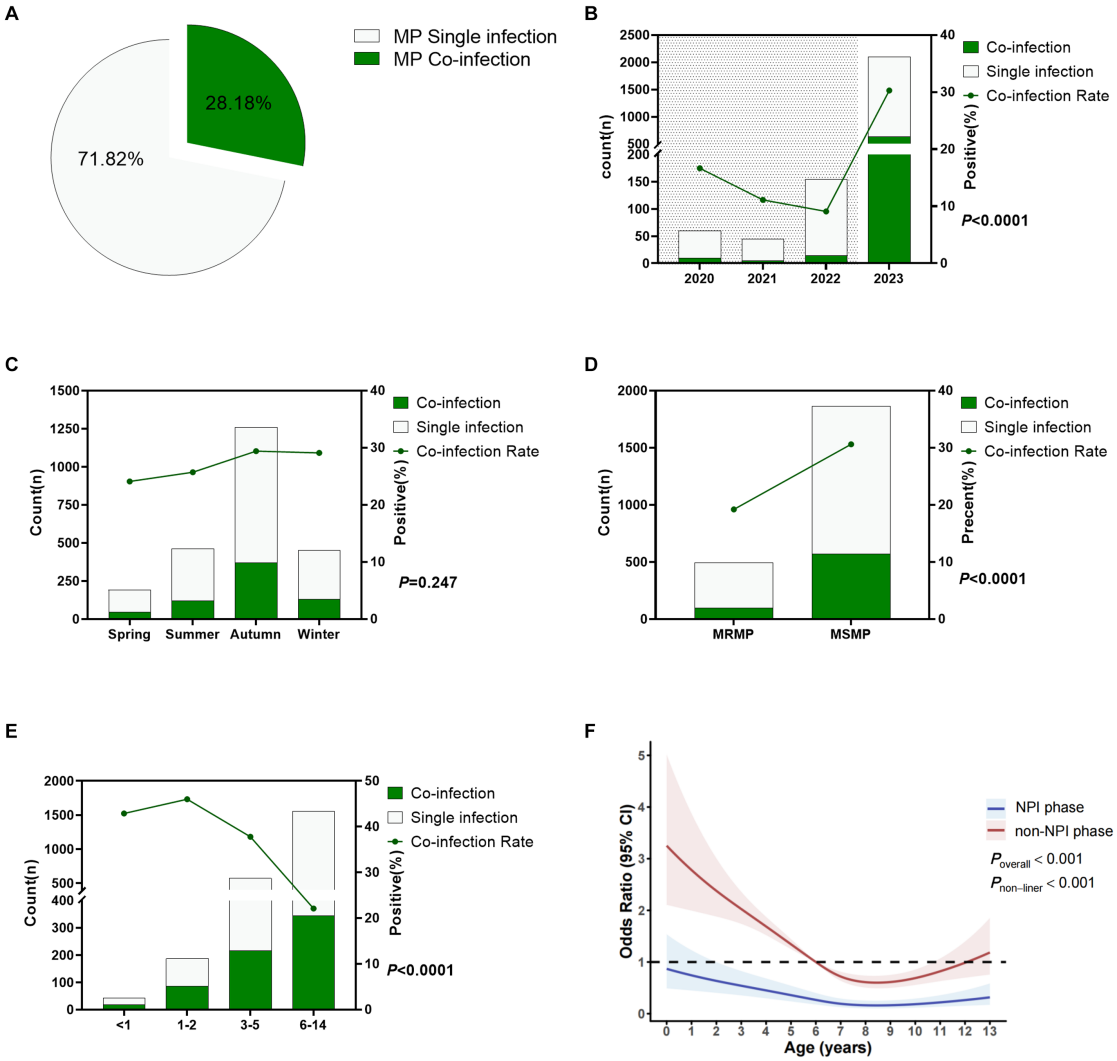
Co-infection status of *Mycoplasma pneumoniae*. **(A)** Overall MP co-infection rate. **(B)** MP co-infection rates in different years. Shaded areas represent the NPI phase, while blank areas represent the non-NPI phase. *p*-values indicate the difference in MP co-infection rates between the NPI and non-NPI phases. **(C)** MP co-infection rates in different seasons. **(D)** Difference in MP co-infection rates between MRMP and MSMP. **(E)** MP co-infection rates in different age groups. **(F)** Nonlinear analysis of the age-related risk of MP co-infection, with colored shadows representing the 95% confidence interval of the odds ratio (OR). Light green represents the number of cases with MP single infection, while dark green represents cases with MP co-infection **(A–E)**.

Through the detection of 13 respiratory pathogens, any positive result in addition to MP was considered as MP co-infection. Among the 2,360 cases of MP-positive patients, 665 cases had MP co-infections, with a co-infection rate of 28.18%. Details are shown in Figure 4A. Figure 4B shows that the MP co-infection rates from 2020 to 2023 were 10.67, 11.11, 9.09, and 30.27%, respectively. The MP co-infection rate in the NPI phase was 11.2%, significantly lower than the rate of 30.3% in the non-NPI phase (*p* < 0.0001). Figure 4C shows that there was no significant difference in the MP co-infection rate among the four seasons, with rates ranging from 24.1 to 29.4%. The MP co-infection rate differed significantly between the macrolide-resistant and sensitive groups (*p* < 0.0001), with a co-infection rate of 19.2% in the resistant group, significantly lower than the rate of 30.6% in the sensitive group (Figure 4D). Figure 4E indicates that the co-infection rates in children aged <1 year, 1–2 years, 3–5 years, and 6–14 years were 42.9, 46.0, 37.8, and 22.1%, respectively, with significant differences among age groups (*p* < 0.0001). Non-restricted cubic spline analysis revealed that the risk factors in the non-NPI phase were higher than those in the NPI phase (Figure 4F). In the NPI phase, all age groups were not susceptible to MP co-infection, while in the non-NPI phase, children aged 0–6 years were susceptible to MP co-infection. Eight years old was a turning point for MP co-infection. For children under 8 years old, the MP co-infection rate decreased with age, while for patients over 8 years old, the MP co-infection rate increased with age.

### 3.6 Interaction analysis between MP and other pathogens

**Figure 5:**
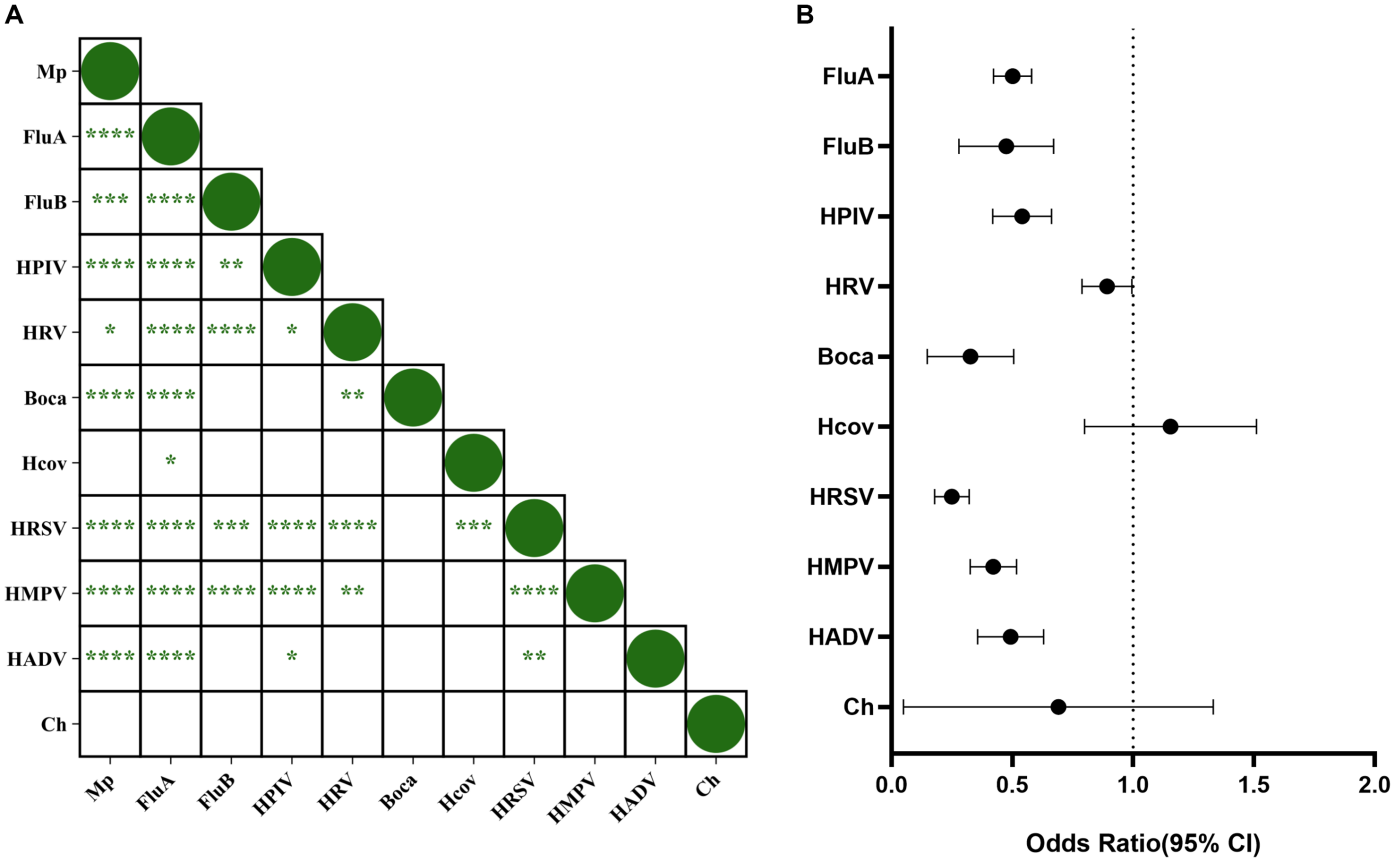
Correlation between *Mycoplasma pneumoniae* and other respiratory pathogens. **(A)** Correlation between respiratory pathogens. *p*-values within each square indicate the correlation between the respective pathogens. “****” represents *p* < 0.0001, “***” represents *p* < 0.001, “*” represents *p* < 0.05, and blank indicates no statistically significant difference between the two groups. **(B)** Interaction between MP and other pathogens. The origin and horizontal lines represent the odds ratio and 95% confidence interval of the interaction between each pathogen and MP. If the horizontal line intersects with 1, it indicates no interaction between the two pathogens. If it is less than 1, it indicates antagonism, while greater than 1 indicates promotion.

To study the correlation between respiratory pathogens, we conducted an interaction analysis (Figure 5A). The results showed that MP interacted with FluA, FluB, HPIV, HRV, Boca, HRSV, HMPV, and HADV, while no interaction was observed between MP and Hcov or Ch. FluA, FluB, HRSV, and HMPV also interacted with various pathogens. Figure 5B shows the odds ratio (OR) of the interaction between MP and various pathogens, revealing antagonistic interactions between MP and FluA, FluB, HPIV, HRV, Boca, HRSV, HMPV, and HADV.

### 3.7 Infection distribution of MP, MRMP, and MP co-infection cases

**Figure 6:**
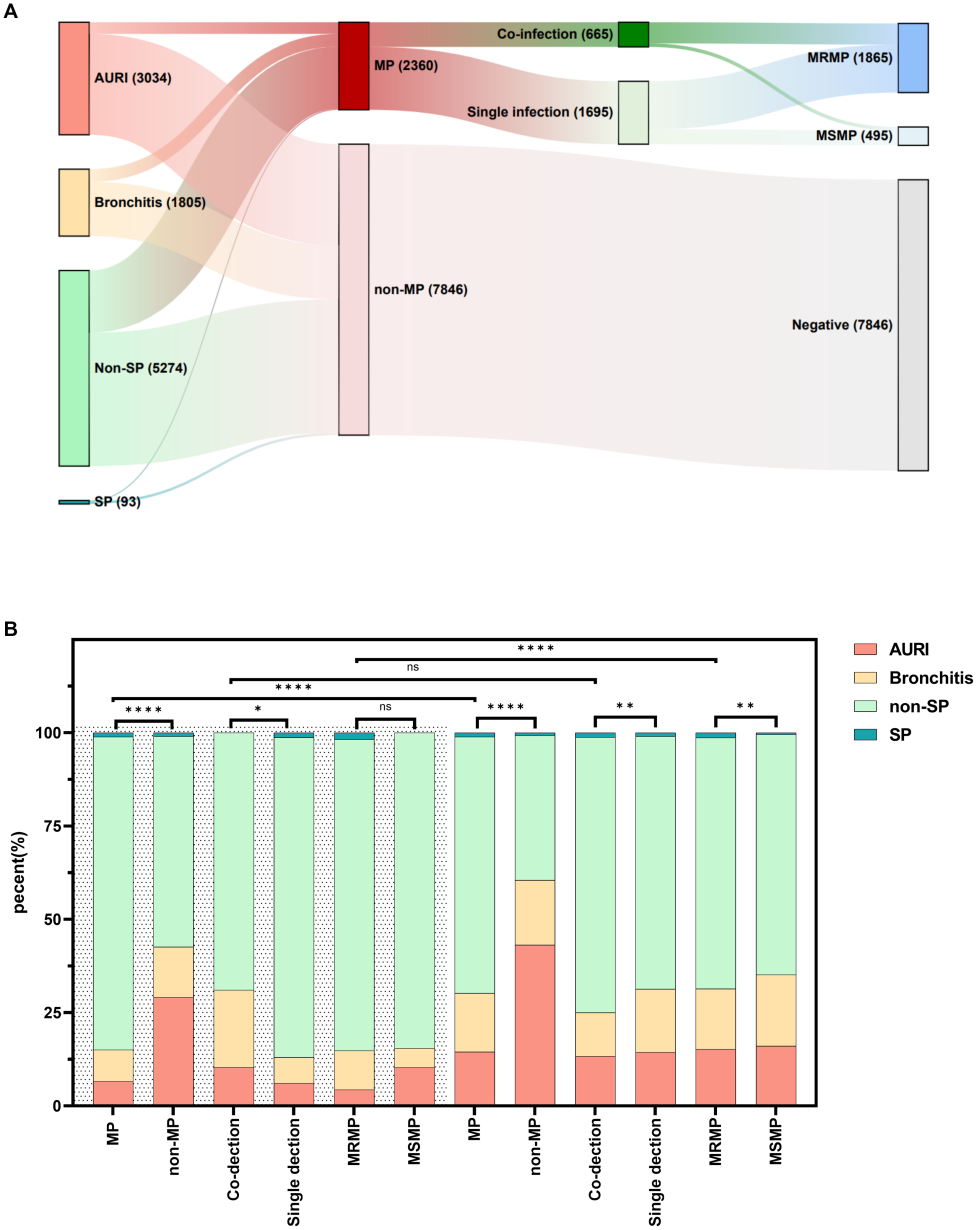
Distribution of diseases in children infected with *Mycoplasma pneumoniae*, macrolide-resistant *Mycoplasma pneumoniae*, and MP co-infection. **(A)** Distribution of MP, MRMP, and MP co-infection in different diseases. **(B)** Distribution of diseases in children infected with MP, MRMP, and MP co-infection. “****” represents *p* < 0.0001, “**” represents *p* < 0.01, “*” represents *p* < 0.05, and “ns” represents no statistically significant difference between the two groups.

This study included a total of 10,206 ARTI patients in Ningbo, among which there were 3,034 cases of upper respiratory infection (URTI), 1,805 cases of bronchitis, 5,274 cases of non-severe pneumonia (non-SP), and 93 cases of severe pneumonia (SP). Details are shown in Figure 6A. The detection rates of MP varied among different types of diseases (*p* < 0.0001), with the highest detection rate in non-severe pneumonia at 31.8%, followed by 28.0% in severe pneumonia, 19.1% in bronchitis, and the lowest at 10.3% in upper respiratory tract infections.

During the NPI phase, there were differences in the clinical diagnosis distribution of MP, MP co-infection, and single infection compared to non-MP cases (*p* < 0.001). The proportion of pneumonia was higher in MP and MP co-infection cases, while the clinical symptom distribution did not differ significantly between MRMP and MSMP cases. In the non-NPI phase, there were differences in the clinical diagnosis distribution among MP, MP co-infection, and single infection compared to non-MP cases (*p* < 0.001). The proportion of pneumonia was higher in MP, MP co-infection, and MRMP cases. There were differences in the clinical diagnosis distribution of MP and MRMP cases between the two phases (*p* < 0.001), with a higher proportion of pneumonia cases in MP and MRMP cases during the non-NPI phase. However, the distribution of clinical diagnoses in co-infection cases did not differ between the two phases. See details in Figure 6B.

## 4 Discussion

Since January 2020, Ningbo has implemented NPIs to curb the spread of COVID-19. The implementation of NPIs has not only effectively controlled the transmission of severe acute respiratory syndrome coronavirus 2 (SARS-CoV-2) but also had a significant impact on the prevalence of respiratory pathogens. In December 2022, China announced further optimization of COVID-19 epidemic prevention and control measures, gradually easing NPIs. This signifies China’s transition into a new phase of the post-COVID-19 pandemic era starting from 2023. This study provides the first assessment of the prevalence characteristics of MP among children in Ningbo during the COVID-19 pandemic, both before and after the relaxation of NPIs, as well as changes in its relationship with other respiratory pathogens and co-infection rates.

This study included a total of 10,206 pediatric patients. The results revealed no significant difference in gender among patients with MP infection. Hospitalization as a surrogate indicator for severe illness, hospitalization numbers for MP-positive patients were lower than outpatient cases, and the MP positivity rate among hospitalized patients was significantly lower than among outpatients, indicating that MP infection often exhibits self-limiting characteristics, consistent with studies by Choi et al. (2019) and Zheng et al. (2021). During the implementation of NPIs, the number of ARTI patients remained around 150 per month, with an MP positivity rate of 6.35%. However, after the relaxation of NPI measures, the number of ARTI patients surged monthly, with an MP positivity rate reaching 34.28%. This increase may be related to measures such as mandatory mask-wearing, maintaining social distancing, and reducing interpersonal transmission (Chow et al., 2023), or it may be associated with antigenic shift and increased population susceptibility (Meyer Sauteur and Beeton, 2023). Liu et al. (2024) believed that the immunity gap (the waning of humoral immunity) was an important influencing factor. During the prolonged 3-year period of NPI implementation, due to insufficient pathogen stimulation, herd immunity decreased. Therefore, after the lifting of epidemic prevention and control measures, the relatively weakened immune system cannot resist the invasion of MP pathogens, leading to an outbreak of MP infection. This phenomenon has also been observed with various respiratory pathogens such as influenza viruses, human respiratory syncytial virus, human coronaviruses, human parainfluenza viruses, and human metapneumovirus (Park et al., 2021; Takashita et al., 2021; Yum et al., 2021; Zhang et al., 2021; Chow et al., 2023).

MP infection exhibits clear seasonality, with the highest prevalence observed in autumn, followed by summer and the lowest in spring. Previous studies have confirmed this phenomenon (Tian et al., 2017). It was found that the optimal growth temperature for MP is 36–37°C, and the MP infection rate gradually increases with the rise in the lowest temperature. Summer and autumn are the hottest months of the year, and higher temperature conditions favor MP replication. During the NPI period, effective control measures kept the MP positivity rate consistently low, and seasonality was not obvious. However, with the relaxation of NPIs, the seasonality of MP infection became more pronounced.

The median age of the included pediatric patients in this study was 4 years, while the median age of MP-infected patients was 6 years. The MP infection rate varied among different age groups, with the highest rate observed in the school-age group, followed by the preschool group, and the lowest in the infant group. Studies by Guo et al. (2019), Liu et al. (2020), and Jiang et al. (2023) have all confirmed this result. The higher MP infection rate in school-age children may be due to reduced social distancing and increased cross-infection (Cai et al., 2022). To further analyze the relationship between MP infection risk and age during the two phases, we used cubic spline regression models. The results showed that during the NPI phase, all age groups were less susceptible to MP, while during the non-NPI phase, the age range of 4–12 years was the most susceptible to MP, with 8-year-old children being the most susceptible. MP infection rates increased with age in children under 8 years old, while they decreased with age between 8 and 14 years old. Wang et al. (2022) study also found a higher detection rate of MP among patients in the middle age group and lower rates at both ends. The exact reasons for this specific age distribution are not fully understood, but it may be due to the continuous maturation of the children’s immune system and the gradual weakening of maternal immunity, which mutually constrain each other (Kloc et al., 2020).

The resistance of *Mycoplasma pneumoniae* (MP) to macrolides has been steadily increasing worldwide, with particularly severe situations in Asian countries. MRMP rates in Japan have risen from 5% in 2002 to 87% in 2014 (Morozumi et al., 2010; Principi and Esposito, 2013; Zhou et al., 2015), while several studies in China have shown an increase in MP resistance from 63 to 92% (Liu et al., 2010; Zhou et al., 2015). However, during the COVID-19 pandemic, Chen et al. (2022) found a decrease in MP resistance, which is consistent with our study. In our research, the macrolide resistance rate during the NPI phase (62.5%) was significantly lower than during the non-NPI phase (81.1%). This may be due to reduced infections of various pathogens and decreased macrolide usage during the NPI phase (Chow et al., 2023). With the outbreak of MP infections, macrolides as first-line treatment drugs have seen a surge in MP resistance rates. The extremely high macrolide resistance rate poses significant challenges to clinical treatment, necessitating an urgent need for appropriate antibiotics to replace macrolides to combat MP infections.

Previous studies have reported viral interference among pathogens (Chow et al., 2023), and our study found that MP interacts antagonistically with FluA, FluB, HPIV, HRV, Boca, HRSV, HMPV, and HADV. The generation of this interference mechanism may have several reasons. Firstly, pathogen infections stimulate the human body’s innate immune system, leading to the release of interferons, which may inhibit the infection of other pathogens (Essaidi-Laziosi et al., 2020). Secondly, due to limited host resources, there may be competition among various pathogens (Shinjoh et al., 2000). Additionally, some studies have found that infection by certain pathogens may inhibit the entry of other pathogens into host cells (Huang et al., 2008). Although MP exhibits antagonistic effects with various respiratory pathogens, cases of MP co-infection with other respiratory pathogens are still observed. A multicenter study in Beijing showed a co-infection rate of up to 27.1% for MP (Wang et al., 2022). In this study, one or more other respiratory pathogens were detected in 28.18% (665/2,360) of MP-positive specimens. It is noteworthy that the co-infection rate during the NPI phase was significantly lower than during the non-NPI phase, with co-infection being more common in MSMP, and the age group of 0–6 years being susceptible to MP co-infections.

Our study found that MP-induced pneumonia accounted for 31.73% (1,703/5,367) of community-acquired pneumonia cases, making it a significant component of community-acquired pneumonia, consistent with the findings of Xu et al. (2024). Chen et al. (2020) found that compared to patients infected with macrolide-sensitive *Mycoplasma pneumoniae* (MSMP), those with macrolide-resistant *Mycoplasma pneumoniae* had a longer duration of fever. Additionally, Chen et al. (2024) pointed out that MP co-infection is a risk factor for community-acquired severe pneumonia. To further understand the impact of MRMP and MP co-infection on the disease, we explored the clinical diagnostic differences among patients infected with MRMP, MSMP, MP single-infection, and co-infection. The results showed that the proportion of pneumonia was higher in MP co-infection patients than in MP single infection patients. MRMP-infected patients had a higher proportion of pneumonia than MSMP-infected patients during the non-NPI phase but not significantly during the NPI phase, possibly due to fewer MP-infected patients during this phase and a significant difference between MRMP and MSMP-infected patients.

It must be acknowledged that this study has certain limitations. Firstly, as it is a retrospective study, some children with acute respiratory tract infection symptoms did not undergo testing for *Mycoplasma pneumoniae* and macrolide resistance as well as the 13 respiratory pathogens, and therefore were not included in the study. Secondly, we only included mutations at positions A2063G and A2064G in domain V of the 23S rRNA in our study, which may underestimate the macrolide-resistance rate. Furthermore, due to the fact that the 13 respiratory pathogens and the macrolide-resistance of *Mycoplasma pneumoniae* in our hospital were scheduled to commence in 2020, there was a lack of pre-epidemic information. Several studies have indicated a decrease in the positive rate of MP during the implementation of NPIs (Cheng et al., 2022; Meyer Sauteur et al., 2022). To enhance the credibility of the current study, Supplementary material including results of *Mycoplasma pneumoniae* specific-IgM antibody tests (colloidal gold method) from 2019 to 2023 were included. The findings revealed that the positive rate of MP was at its lowest during the implementation of NPIs, consistent with PCR results.

## 5 Conclusion

This study provides the first analysis of the epidemiological characteristics of *Mycoplasma pneumoniae* during the NPI phase of the COVID-19 pandemic and the post-epidemic period following the lifting of control measures in Ningbo, China. The removal of NPIs significantly impacted the spread of MP, altering population characteristics including age, seasonality, macrolide resistance, and MP co-infection rates.

## Data availability statement

The original contributions presented in the study are included in the article/Supplementary material, further inquiries can be directed to the corresponding authors.

## Ethics statement

The studies involving humans were approved by Ethics Committee of the Ningbo Medical Center LiHuiLi Hospital (Ethical approval number: KY2024SL070-01). The studies were conducted in accordance with the local legislation and institutional requirements. Written informed consent for participation was not required from the participants or the participants’ legal guardians/next of kin in accordance with the national legislation and institutional requirements.

## Author contributions

MJ: Writing – original draft, Visualization, Software, Methodology, Investigation, Formal analysis, Data curation, Conceptualization. HZ: Writing – review & editing, Methodology, Investigation, Formal analysis, Data curation, Conceptualization. FY: Writing – original draft, Methodology, Data curation. QLu: Writing – original draft, Methodology, Formal analysis, Data curation. QS: Writing – review & editing, Visualization, Software, Methodology, Data curation. ZL: Writing – original draft, Funding acquisition, Data curation. QLi: Writing – review & editing, Funding acquisition, Formal analysis. GW: Writing – review & editing, Methodology, Funding acquisition.
