# Supplementary material for: Influence of non-pharmaceutical interventions on epidemiological characteristics of Mycoplasma pneumoniae infection in children during and after the COVID-19 epidemic in Ningbo, China
Source: Front Microbiol. 2024 Jul 17;15:1405710. doi: 10.3389/fmicb.2024.1405710 (PMC11288959; doi:10.3389/fmicb.2024.1405710)
Supplement: Supplementary file 1 [file Data_Sheet_1.pdf]

Supplemental material

Detection of *Mycoplasma pneumoniae* specific-IgM antibodies in children, 2019-2023

1 Methods

Clinical data of children aged 0-14 years with acute respiratory symptoms (such as cough, rhinorrhea, fever, auscultatory rales in the lungs, wheezing, with or without moist rales) admitted to the Ningbo Medical Center LiHuiLi Hospital from January 2019 to December 2023 were collected. All children underwent testing for *Mycoplasma pneumoniae* specific-IgM antibody (Colloidal gold method, Sanming Bofeng Biotechnology, Sanming, China). Repeat test results are recorded only once within three months. Patients with underlying diseases (congenital diseases, immunodeficiency diseases, etc.), recurrent chronic respiratory diseases, or incomplete clinical data were excluded from the study. This study was divided into three phases: pre-epidemic phase(2019), NPI phase (2020-2022), and non-NPI phase (2023). Other data grouping and processing were consistent with the PCR method.

2 Results

A total of 6,844 cases of acute respiratory tract infection (ARTI) patients were included in this study, with 2,710 cases (39.60%) in the pre-epidemic phase (2019), and 3,489 cases (50.98%) in the NPI phase (2020-2022), and 645 cases (9.42%) in the non-NPI phase (2023). The decline in cases in 2023 can be attributed to the occurrence of multiple respiratory pathogen infections, particularly in children who were more likely to undergo testing for multiple pathogens using a combination of PCR methods. Analysis of Supplementary Table 1 revealed that the NPI phase exhibited the lowest positivity rate for MP ($P<0.001$), regardless of whether the IgM assay or PCR method was utilized. Furthermore, the inclusion of the IgM assay data from the pre-epidemic phase (2019) indicated that the MP positivity rate was highest in the non-NPI phase (36.00%), followed by the pre-epidemic phase implementation (17.82%), and lowest during the NPI phase (9.52%). Based on Supplementary Figure 1, it was evident that the positivity rate of MP per month exhibited greater consistency between the IgM and PCR methods.

The positivity rate of MP varied across age groups in distinct phases, with the highest positivity rate observed during the non-NPI phase and the lowest during the NPI phase. Similarly, variations in MP positivity rates were observed across seasons, with the highest rates occurring during the non-NPI phase and the lowest during the NPI phase, demonstrating consistent patterns within each season.

Supplementary Table1

General clinical characteristics of MP infection at different phases

**Characteristics**	**pre-epidemic phase**	**NPI phase**	**non-NPI phase**	***P*-Vaule**
	**MP Positive**	**MP Positive**	**MP Positive**	
**Method**				
IgM	483(17.82%)	332(9.52%)	232(36.00%)	<0.0001
PCR	-	259(6.35%)	2101(34.28%)	<0.0001
**Group(IgM)**				
<1 year	-	2(9.1%)	-	-
1-2 years	79(13.1%)	49(6.3%)	9(37.5%)	<0.0001
3-5 years	186(15.7%)	133(8.2%)	38(33.9%)	<0.0001
6-14 years	218(23.6%)	148(13.8%)	179(35.2%)	<0.0001
**Season(IgM)**				
Spring	125(16.3%)	73(9.1%)	22(36.7%)	<0.0001
Summer	145(22.5%)	74(8.6%)	26(41.3%)	<0.0001
Autumn	66(11.0%)	79(8.3%)	84(29.0%)	<0.0001
Winter	147(21.0%)	106(12.0%）	94（40.5%）	<0.0001

Note: The rest was used as Pearson's Chi-squared test, and the data was expressed as n (%).

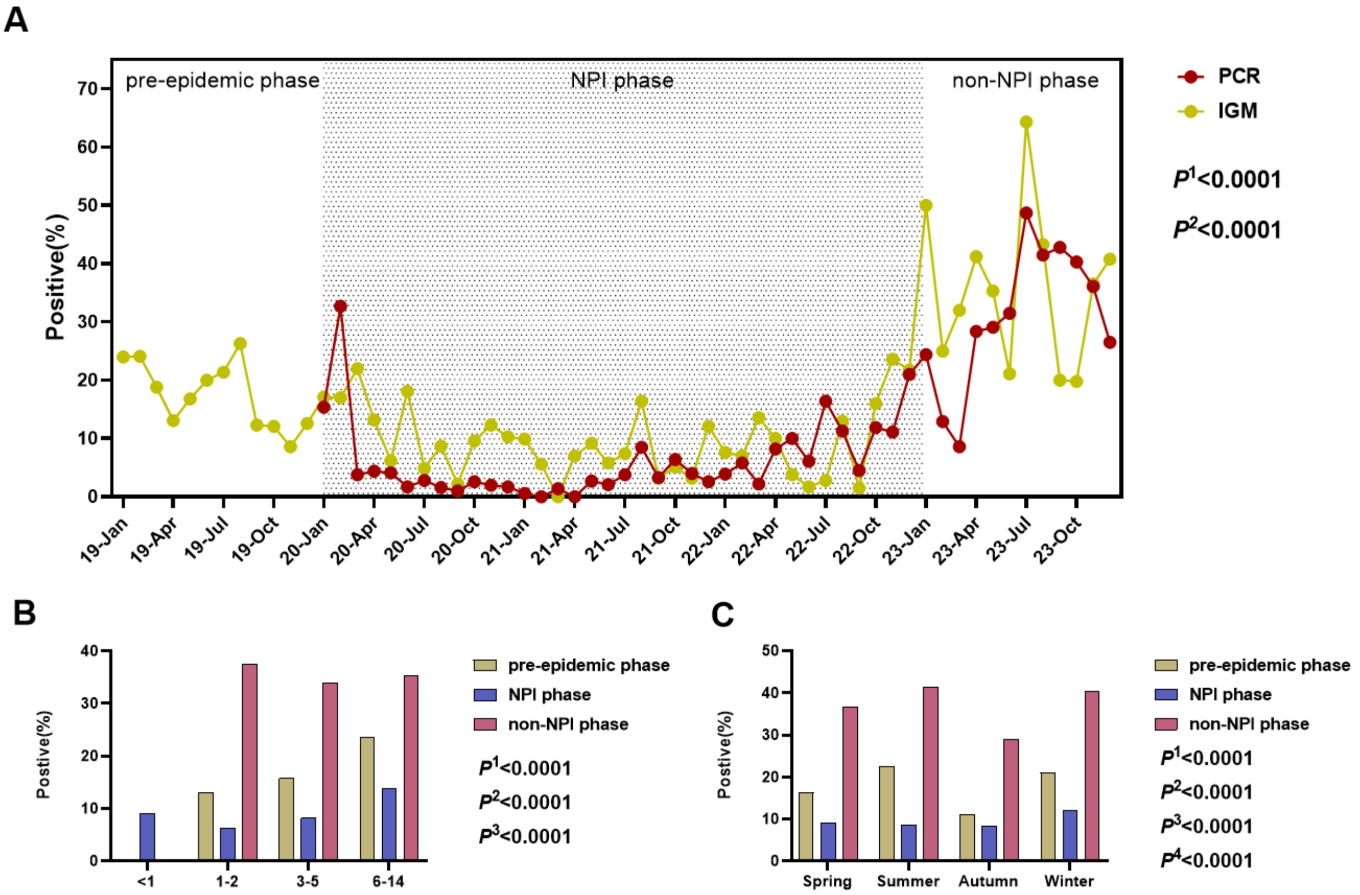

Supplementary Figure 1. General clinical characteristics of *Mycoplasma pneumoniae* (MP) infection at different phases. (A) The monthly positive rate of MP detection by PCR and IgM. P^1 indicates the difference in MP positive rate detected by PCR at different phases. P^2 indicates the difference in MP positive rate in different phases detected by IgM method. (B) The difference of MP positive rate in different ages (IgM). There were no children < 1 year during either the pre-epidemic phase or non-NPI phase. P^1, P^2, and P^3 showed the difference in MP positive rate in different phases of 1-2 years, 3-5 years, and 6-14 years, respectively. (C) The difference of MP positive rate in different seasons (IgM). P^1, P^2, P^3, and P^4 respectively indicate the difference in MP positive rate in different phases in spring, summer, autumn, and winter.
